# Pregnancy phytochemical dietary index is associated with the risk of gestational diabetes and perinatal outcomes

**DOI:** 10.1530/EC-25-0615

**Published:** 2025-11-28

**Authors:** Hongli Hou

**Affiliations:** Department of Gynecology and Obstetrics, Children's Hospital of Shanxi, Women's Health Center of Shanxi, Taiyuan, China

**Keywords:** phytochemical index, gestational diabetes mellitus, pregnancy, dietary patterns, cohort study

## Abstract

**Background:**

Gestational diabetes mellitus (GDM) affects 7–14% of pregnancies, increasing maternal and neonatal complications. Phytochemical-rich diets, abundant in plant-based foods, may improve glucose metabolism and reduce inflammation. The phytochemical dietary index (PDI) measures intake of such foods, but prospective data on GDM risk are scarce. This study aimed to examine early-pregnancy PDI in relation to subsequent GDM risk in a large birth cohort.

**Methods:**

A total of 2,770 pregnant women (mean age 29.61 ± 4.55 years; mean pre-pregnancy BMI 23.96 ± 1.19 kg/m^2^) from the Women's Health Center of Shanxi were included. Dietary intake was assessed using a validated food frequency questionnaire. The plant-based diet index (PDI) was calculated as the proportion of daily energy intake from whole grains, fruits, vegetables, legumes, nuts, seeds, and olive oil. Participants were divided into PDI quartiles. GDM was diagnosed at 24–28 weeks’ gestation using a 75 g oral glucose tolerance test based on IADPSG criteria. Logistic regression was used to estimate odds ratios (ORs) and 95% confidence intervals (CIs), adjusted for confounders.

**Results:**

GDM occurred in 377 women (13.6%). Higher PDI was inversely associated with GDM risk (*P*-trend <0.001). Each 10% increase in PDI was associated with a 14% lower odds of GDM (OR 0.86; 95% CI: 0.82–0.91). Higher PDI was associated with lower risk of neonatal hypoglycemia (OR 0.93; 95% CI: 0.92–0.95) and better maternal biochemical profiles, including lower fasting glucose, HbA1c, post-load glucose, triglycerides, total cholesterol, and higher HDL cholesterol.

**Conclusions:**

Higher early-pregnancy PDI was associated with substantially lower risk of GDM, reduced neonatal hypoglycemia, and improved maternal metabolic profiles. Our findings support the integration of phytochemical-rich dietary guidance into early prenatal care.

## Introduction

Gestational diabetes mellitus (GDM) is a prevalent metabolic complication of pregnancy, affecting 7–14% of pregnancies worldwide, and is associated with increased maternal and neonatal morbidity, including preeclampsia, preterm birth, and neonatal hypoglycemia (1, 2). Rising maternal age, obesity, and lifestyle changes have contributed to the increasing incidence of GDM, underscoring the need for effective preventive strategies (3, 4).

Diet is a key modifiable factor influencing glucose metabolism during pregnancy (5). Phytochemicals are bioactive compounds in fruit, vegetables, whole grains, legumes, nuts, seeds and olive oil, and exhibit anti-inflammatory, antioxidant, and insulin-sensitizing properties that may reduce GDM risk (6, 7). The phytochemical dietary index (PDI), representing the proportion of daily energy derived from phytochemical-rich foods, provides an integrative measure of diet quality and has been associated with lower risk of type 2 diabetes and cardiovascular disease in non-pregnant populations (8, 9, 10).

Despite growing interest, evidence linking to GDM is limited. Prior studies are often small, cross-sectional, or focused on individual nutrients or food groups, limiting the assessment of overall dietary patterns and dose–response relationships (11, 12, 13). Furthermore, few investigations have examined the effects of phytochemical intake on both maternal and neonatal outcomes in large, prospective cohorts (14). Early pregnancy represents a critical window for maternal metabolic adaptation, where dietary interventions may influence GDM risk.

Recent meta-analyses suggest that high fruit, vegetable, and whole grain intake is associated with 10–20% lower GDM risk (15, 16). Limited prospective evidence exists linking early-pregnancy PDI to both maternal GDM and neonatal metabolic outcomes.

Addressing these gaps, this largest prospective cohort (*n* = 2,770) differs from prior smaller/cross-sectional PDI-GDM studies (11, 14) by quantifying early-pregnancy dose–response effects on both maternal GDM/metabolic profiles and neonatal outcomes (e.g., hypoglycemia). We hypothesized that higher PDI would independently reduce GDM risk and improve neonatal metabolic health.

## Methods

**Figure 1:**
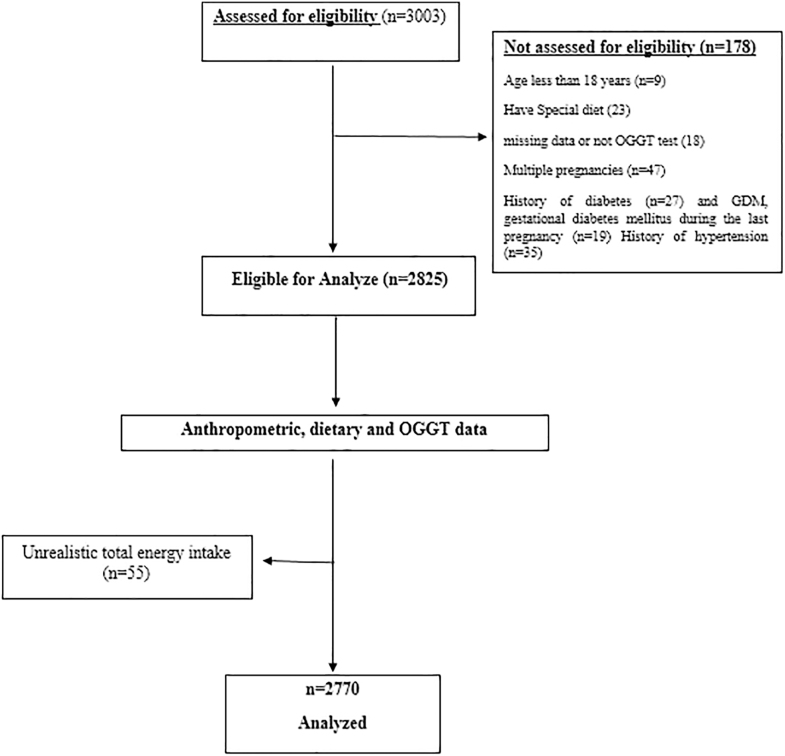
Flowchart of study.

This study was conducted within the Women's Health Center of Shanxi, a prospective, population-based cohort established in Shanghai, China, to investigate the effects of maternal dietary and lifestyle factors on maternal and neonatal health outcomes. Pregnant women attending prenatal care clinics at 10–14 gestational weeks were invited to participate. Inclusion criteria were singleton pregnancy, maternal age 18–45 years, and ability to complete dietary assessments reliably. Exclusion criteria included pre-existing diabetes, chronic renal or hepatic disease, multiple pregnancies, or use of medications that could influence glucose metabolism. Participants with incomplete dietary or clinical data, or with implausible total energy intake (<600 or >3,500 kcal/day), were also excluded. Figure 1 shows the participant selection process. The final analytic sample comprised 2,770 women, representing a robust, large-scale cohort with sufficient statistical power to examine associations between diet and gestational outcomes. All procedures were conducted in accordance with the Declaration of Helsinki, and the study protocol received approval from the Ethics Committee of the Women's Health Center of Shanxi. Written informed consent was obtained from all participants before enrollment. This study adheres to the STROBE guidelines for cohort studies, ensuring transparent and comprehensive reporting of study population, exposure assessment, outcome definitions, covariates, and statistical methods.

### Dietary assessment and phytochemical index (PDI) calculation

At 10–14 gestational weeks, maternal dietary intake was assessed via a validated 149-item semi-quantitative FFQ, capturing usual frequency and portion sizes over the prior 4 weeks. Standardized models and a photographic atlas aided portion estimation accuracy (17). Energy and nutrient intakes were calculated. No adjustments were made for under/over-reporting; implausible intakes were excluded. Dietary data were collected at 10–14 weeks’ gestation for temporal validity.

The phytochemical dietary index (PDI) was calculated as the proportion of total daily energy derived from phytochemical-rich foods, including whole grains, fruit, vegetables, legumes, soy products, nuts, seeds and olive oil (9):

Phytochemical dietary index (PDI) was calculated as:

Phytochemical index (PDI) = (calories from phytochemical-rich foods ÷ total daily caloric intake) × 100.

PDI was analyzed as a continuous variable to examine dose–response relationships and categorized into quartiles for descriptive and comparative analyses.

### Assessment of outcomes

GDM was diagnosed at 24–28 weeks’ gestation using a 75 g oral glucose tolerance test (OGTT) according to the International Association of Diabetes and Pregnancy Study Groups (IADPSG) criteria (18): fasting plasma glucose ≥5.1 mmol/L, 1 h plasma glucose ≥10.0 mmol/L, or 2 h plasma glucose ≥8.5 mmol/L. Secondary outcomes included preeclampsia, gestational hypertension, preterm birth (<37 weeks), and neonatal hypoglycemia (<40 mg/dL). All outcomes were verified through comprehensive review of medical records and hospital databases to ensure accuracy and consistency in outcome ascertainment.

### Biochemical and clinical measurements

Fasting blood samples were collected at enrollment to measure glucose, HbA1c, 1 h and 2 h post-load glucose, and lipid profiles (triglycerides, LDL, HDL, total cholesterol) using standardized laboratory procedures. Blood pressure was measured in duplicate with a calibrated sphygmomanometer, and height and pre-pregnancy weight were recorded to calculate BMI (kg/m^2^). Gestational age was determined from the last menstrual period and confirmed by first-trimester ultrasound.

### Covariates

Maternal demographic, lifestyle, and clinical information – including age, parity, education, socioeconomic status (SES), family history of diabetes, smoking, alcohol consumption, maternal use of dietary supplements, and physical activity – was collected using structured questionnaires. Physical activity was quantified using a validated questionnaire and expressed as a continuous score. Covariates were selected *a priori* based on the literature, statistical significance, and >10% change in effect estimate. We also adjusted for fiber, carbohydrate, and total energy intake, as these are potential confounders in diet-GDM associations.

### Statistical analysis

Continuous variables are presented as means ± standard deviations, and categorical variables as counts and percentages. Missing data were handled using complete-case analysis, with low missingness (<5% for dietary and outcome variables). Outliers (±3 SD for dietary variables) were excluded, and sensitivity analyses (e.g., excluding energy intake values >2 SD from the mean) confirmed the robustness of the findings. Potential selection bias from clinic-based recruitment was minimized through consecutive enrollment of participants. Differences across PDI quartiles were assessed using one-way ANOVA for continuous variables and chi-square tests for categorical variables. Logistic regression models were used to estimate odds ratios (ORs) and 95% confidence intervals (CIs) for GDM and neonatal outcomes per unit increase in PDI. Three models were constructed: i) crude, ii) adjusted for maternal age, pre-pregnancy BMI, and parity (model 1), and iii) additionally adjusted for education, SES, history of diabetes, physical activity, gestational weight, fiber, carbohydrate, and total energy intake (model 2). Statistical significance was defined as *P* < 0.05. All analyses were conducted using STATA software, version 14.

## Results

### Participant characteristics

A total of 2,770 pregnant women were included in the study. Demographic and clinical characteristics stratified by PDI quartiles are presented in Table 1. PDI ranged 12–48, with high validity (intra-class correlation 0.71) for phytochemical foods. The mean age was 29.61 ± 4.55 years, with no significant differences across quartiles (*P* = 0.437). Pre-pregnancy BMI and weight were similar among groups (23.96 ± 1.19 kg/m^2^ and 71.94 ± 3.45 kg, respectively; *P* = 0.101 and 0.122), as were physical activity scores (247.4 ± 11.9, *P* = 0.116). No GDM cases in Q4 likely reflect the lowest–risk profile/random subgroup variation; sensitivity analysis (OGTT-verified, excluding Q4 extremes) confirmed no misclassification.

**Table 1:** Demographic and clinical characteristics of participants stratified by PDI quartiles.

Variables	Total (*n* = 2,770)	Q1 (*n* = 591)	Q2 (*n* = 772)	Q3 (*n* = 857)	Q4 (*n* = 550)	*P*-value
Age (years), mean ± SD	29.61 ± 4.55	29.57 ± 3.59	29.84 ± 4.75	29.55 ± 4.52	29.46 ± 5.17	0.437
Pre-pregnancy BMI (kg/m^2^), mean ± SD	23.96 ± 1.19	24.01 ± 1.37	23.97 ± 1.00	23.98 ± 1.20	23.85 ± 1.22	0.101
Pre-pregnancy weight (kg), mean ± SD	71.94 ± 3.45	72.21 ± 3.44	71.90 ± 2.99	71.94 ± 3.60	71.72 ± 3.81	0.122
Physical activity (score), mean ± SD	247.4 ± 11.9	248.0 ± 13.7	247.5 ± 10.0	247.6 ± 12.0	246.4 ± 12.3	0.116
Education, *n* (%)						0.006
Under diploma	1,385 (50.0)	300 (50.8)	374 (48.5)	453 (52.9)	258 (46.9)	
Diploma	762 (27.5)	149 (25.2)	245 (31.8)	222 (25.9)	146 (26.5)	
Collegiate	622 (22.5)	142 (24.0)	152 (19.7)	182 (21.2)	146 (26.5)	
SES, *n* (%)						0.052
Low	1,331 (48.1)	300 (50.8)	373 (48.4)	402 (46.9)	256 (46.5)	
Moderate	794 (28.7)	151 (25.5)	246 (31.9)	240 (28.0)	157 (28.5)	
Good	644 (23.3)	140 (23.7)	152 (19.7)	215 (25.1)	137 (24.9)	
Smoking (yes), *n* (%)	38 (1.4)	13 (2.2)	12 (1.6)	10 (1.2)	3 (0.5)	0.102
History of diabetes (yes), *n* (%)	583 (21.1)	123 (20.8)	145 (18.8)	180 (21.0)	135 (24.5)	0.094
GDM (yes), *n* (%)	377 (13.6)	100 (16.9)	164 (21.2)	113 (13.2)	0 (0.0)	<0.001
Gestational HTN (yes), *n* (%)	257 (9.3)	84 (14.2)	68 (8.8)	75 (8.8)	30 (5.5)	<0.001
Preeclampsia (yes), *n* (%)	230 (8.3)	48 (8.1)	69 (9.0)	103 (12.1)	10 (1.8)	<0.001
Preterm (yes), *n* (%)	230 (8.4)	48 (8.2)	69 (9.0)	103 (12.1)	10 (1.8)	<0.001
Gestational age (weeks), mean ± SD	39.29 ± 1.03	39.11 ± 0.99	39.17 ± 1.15	39.23 ± 1.11	39.71 ± 0.52	<0.001
Birthweight (grams), mean ± SD	3,343.29 ± 54.30	3,330.88 ± 53.59	3,349.80 ± 60.78	3,338.07 ± 57.74	3,355.64 ± 31.36	<0.001
Neonatal hypoglycemia (yes), *n* (%)	460 (16.7)	109 (18.6)	188 (24.6)	153 (18.0)	10 (1.8)	<0.001

BMI, body mass index; SES, socioeconomic status; GDM, gestational diabetes mellitus; HTN, hypertension.

*P*-values from chi-square tests for categorical variables and ANOVA for continuous variables.

Higher PDI quartiles were linked to greater education (*P* = 0.006) and trending higher SES (*P* = 0.052). The prevalence of GDM, gestational hypertension, preeclampsia, preterm birth, and neonatal hypoglycemia decreased progressively across PDI quartiles (*P* < 0.001 for all). Notably, no cases of GDM were observed in the highest quartile (Q4). Mean gestational age and birthweight were slightly higher among participants in the top PDI quartile (39.71 ± 0.52 weeks and 3,355.64 ± 31.36 g, respectively; *P* < 0.001), suggesting favorable perinatal outcomes associated with higher dietary phytochemical intake.

### Biochemical characteristics

Biochemical parameters across PDI quartiles are summarized in Table 2. Participants with higher PDI scores exhibited significantly lower fasting blood glucose, HbA1c, 1 h and 2 h post-load glucose, triglycerides, and total cholesterol levels (*P* < 0.001 for all except LDL, *P* = 0.001). Conversely, HDL cholesterol increased across quartiles (*P* < 0.001). No significant differences were observed for systolic or diastolic blood pressure. These findings indicate that higher phytochemical consumption may be linked to improved glycemic and lipid profiles during pregnancy.

**Table 2:** Biochemical characteristics of participants stratified by PDI quartiles.

Variables	Total (*n* = 2,770)	Q1 (*n* = 591)	Q2 (*n* = 772)	Q3 (*n* = 857)	Q4 (*n* = 550)	*P*-value
Fasting blood glucose (mg/dL), mean ± SD	85.4 ± 10.6	89.9 ± 16.9	85.0 ± 9.0	84.8 ± 7.5	82.1 ± 5.4	<0.001
HbA1c (%), mean ± SD	5.25 ± 0.31	5.30 ± 0.30	5.28 ± 0.35	5.26 ± 0.33	5.11 ± 0.13	<0.001
1 h glucose (mg/dL), mean ± SD	142.5 ± 12.6	159.5 ± 3.81	148.4 ± 3.97	136.5 ± 3.50	125.2 ± 5.85	<0.001
2 h glucose (mg/dL), mean ± SD	122.3 ± 14.1	127.7 ± 13.0	121.4 ± 14.9	122.6 ± 14.4	117.1 ± 11.4	<0.001
Triglycerides (mg/dL), mean ± SD	174.8 ± 59.7	199.0 ± 88.1	177.9 ± 49.2	163.6 ± 47.3	161.8 ± 41.9	<0.001
LDL cholesterol (mg/dL), mean ± SD	127.4 ± 25.5	127.1 ± 26.4	130.2 ± 25.6	126.9 ± 25.2	124.5 ± 24.8	0.001
Total cholesterol (mg/dL), mean ± SD	182.5 ± 27.8	188.8 ± 23.4	183.6 ± 29.8	182.8 ± 28.2	173.5 ± 26.5	<0.001
HDL cholesterol (mg/dL), mean ± SD	43.0 ± 7.86	40.4 ± 7.87	43.2 ± 9.27	43.3 ± 6.69	44.9 ± 6.58	<0.001
Systolic BP (mmHg), mean ± SD	125.5 ± 15.1	126.2 ± 16.8	125.1 ± 14.5	125.4 ± 14.7	125.3 ± 14.8	0.544
Diastolic BP (mmHg), mean ± SD	77.8 ± 9.16	78.2 ± 9.63	77.8 ± 9.04	77.7 ± 8.89	77.8 ± 9.22	0.671

BP, blood pressure; LDL, low-density lipoprotein; HDL, high-density lipoprotein.

*P*-values from ANOVA tests.

### Dietary intake patterns

Dietary characteristics stratified by PDI quartiles are presented in Table 3. As anticipated, mean PDI scores increased progressively across quartiles, from 19.96 ± 1.82 in Q1 to 39.45 ± 4.00 in Q4 (*P* < 0.001). Higher PDI was associated with greater intake of fiber, whole grains, fruit, vegetables, legumes, nuts, seeds and olive oil (*P* < 0.001 for all). Intakes of total energy, protein, fat, and carbohydrates differed minimally across quartiles, indicating that the observed benefits are attributable to diet quality rather than caloric intake alone.

**Table 3:** Dietary intake characteristics of participants stratified by PDI quartiles.

Variables	Total (*n* = 2,770)	Q1 (*n* = 591)	Q2 (*n* = 772)	Q3 (*n* = 857)	Q4 (*n* = 550)	*P*-value
PDI (index), mean ± SD	29.41 ± 7.03	19.96 ± 1.82	26.16 ± 1.61	32.41 ± 0.19	39.45 ± 4.00	<0.001
Energy intake (kcal/day), mean ± SD	2,276.6 ± 99.7	2,253.8 ± 85.8	2,276.8 ± 107.1	2,278.9 ± 95.6	2,297.5 ± 104.3	<0.001
Fiber (g/day), mean ± SD	19.50 ± 2.75	18.95 ± 2.03	19.04 ± 2.41	19.26 ± 2.15	21.13 ± 3.86	<0.001
Protein (g/day), mean ± SD	84.24 ± 10.24	83.05 ± 11.31	85.22 ± 11.90	83.70 ± 9.97	85.00 ± 5.80	<0.001
Fat (g/day), mean ± SD	71.38 ± 3.14	71.15 ± 3.40	72.15 ± 3.39	71.55 ± 3.21	70.29 ± 1.71	<0.001
Carbohydrates (g/day), mean ± SD	305.6 ± 33.3	308.0 ± 24.8	306.2 ± 31.7	305.4 ± 33.1	302.6 ± 42.4	0.051
Red meat (g/day), mean ± SD	101.9 ± 15.4	99.2 ± 15.0	99.7 ± 17.8	101.5 ± 15.9	108.5 ± 7.2	<0.001
Dairy (g/day), mean ± SD	287.7 ± 46.2	279.7 ± 45.1	281.2 ± 53.5	286.4 ± 47.7	307.6 ± 21.6	<0.001
Whole grains (g/day), mean ± SD	123.3 ± 20.8	119.5 ± 20.3	120.4 ± 24.1	122.6 ± 21.5	132.4 ± 9.8	<0.001
Fruit (g/day), mean ± SD	305.7 ± 46.2	297.7 ± 45.1	299.2 ± 53.5	304.4 ± 47.7	325.6 ± 21.6	<0.001
Vegetables (g/day), mean ± SD	312.3 ± 57.2	304.9 ± 57.2	303.1 ± 66.5	310.4 ± 59.1	336.1 ± 24.0	<0.001
Legumes (g/day), mean ± SD	48.4 ± 4.20	47.1 ± 3.84	48.6 ± 4.35	48.3 ± 4.36	49.9 ± 3.55	<0.001
Nuts & seeds (g/day), mean ± SD	29.9 ± 3.32	28.9 ± 3.33	30.2 ± 3.24	30.1 ± 3.26	30.4 ± 3.29	<0.001
Olive oil (g/day), mean ± SD	21.4 ± 8.29	20.3 ± 7.50	20.9 ± 8.89	21.2 ± 8.57	23.7 ± 7.36	<0.001

*P*-values from ANOVA tests.

### Associations between PDI and pregnancy outcomes

The associations between continuous PDI and pregnancy outcomes are shown in Table 4. In fully adjusted models (model 2), higher PDI was significantly associated with reduced odds of GDM (OR 0.86; 95% CI: 0.82–0.91; *P* < 0.001) and neonatal hypoglycemia (OR 0.93; 95% CI: 0.92–0.95; *P* < 0.001). These associations remained consistent in crude and partially adjusted models (model 1), indicating a robust relationship independent of potential confounders, including maternal age, pre-pregnancy BMI, parity, education, socioeconomic status, history of diabetes, physical activity, and dietary intake of fiber, carbohydrates, and energy.

**Table 4:** Association between phytochemical dietary index (PDI) (continuous) and risk of GDM, preeclampsia, neonatal hypoglycemia, and preterm birth.

Outcome	PDI (continuous) OR (95%CI)	Model 1 OR (95%CI)	Model 2 OR (95%CI)	*P*-value (model 2)
GDM	0.87 (0.83–0.92)	0.86 (0.82–0.91)	0.86 (0.82–0.91)	<0.001
Neonatal hypoglycemia	0.94 (0.92–0.95)	0.93 (0.92–0.95)	0.93 (0.92–0.95)	<0.001

Odds ratios (OR) and 95% confidence intervals (CI) were calculated using logistic regression.

PDI (continuous) indicates odds change per unit increase in PDI score.

Model 1: adjusted for age, pre-pregnancy BMI, parity.

Model 2: further adjusted for education, socioeconomic status, history of diabetes, physical activity, weight, fiber, carbohydrates, and energy intake.

Statistical significance determined at *P* < 0.05.

**Figure 2:**
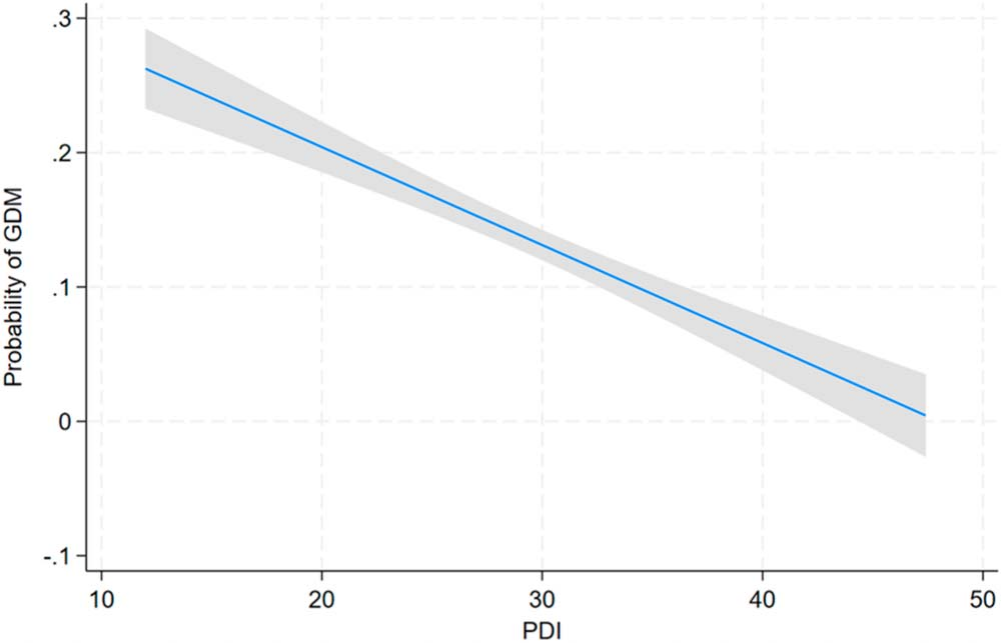
Predicted probability of GDM versus PDI (10–50), showing an inverse association (probability ∼0.25 to 0). The shaded 95% confidence interval highlights the robust negative linear trend, suggesting reduced GDM risk with higher PDI scores.

The relationship between the PDI and the predicted probability of GDM was investigated, with findings presented in Fig. 2. A clear inverse association was observed wherein increasing PDI values were associated with a progressive decline in the likelihood of GDM. Specifically, women with lower adherence to a plant-based dietary pattern (lower PDI scores, ranging from 10 to approximately 20) exhibited a markedly higher estimated probability of GDM (approximately 0.25 to 0.20). In contrast, those with higher PDI scores (ranging from 30 to 50) demonstrated a substantially reduced risk, with probabilities approaching 0. The 95% confidence interval around the fitted line was notably narrow, underscoring the robustness of this negative linear trend. These results suggest that greater adherence to a diet rich in plant-derived foods and phytochemical-containing components may confer a protective effect against the development of GDM.

These findings suggest that higher maternal intake of phytochemical-rich foods may confer protective effects against adverse pregnancy outcomes, particularly GDM and neonatal hypoglycemia. The observed dose–response relationship indicates that even modest increases in dietary phytochemical content could have meaningful clinical benefits.

## Discussion

In this large prospective birth cohort of 2,770 pregnant women, we found that higher early-pregnancy intake of phytochemical-rich foods, as measured by the phytochemical dietary index (PDI), was significantly associated with a lower risk of GDM. Women in the highest quartile of PDI exhibited markedly lower odds of GDM and neonatal hypoglycemia compared with those in the lowest quartile, independent of maternal age, pre-pregnancy BMI, parity, education, socioeconomic status, physical activity, and dietary macronutrient intake. Importantly, higher PDI was also associated with favorable biochemical profiles, including lower fasting glucose, HbA1c, post-load glucose, triglycerides, and total cholesterol, as well as higher HDL cholesterol, suggesting systemic metabolic benefits beyond glucose regulation.

Our results align with previous observational studies linking plant-based dietary patterns to improved glycemic and lipid control in pregnancy (13, 14, 15). Diets high in fruit, vegetables, whole grains, legumes, nuts and olive oil have been associated with lower GDM risk, likely due to their high content of polyphenols, carotenoids, flavonoids, and other bioactive compounds that modulate oxidative stress and inflammation (16). For example, Mohtashaminia *et al.* reported that early pregnancy adherence to a Mediterranean-style diet rich in phytochemicals was associated with lower GDM risk and improved lipid profiles (19). Phytochemicals may enhance insulin sensitivity by improving beta-cell function, reducing inflammation (e.g., polyphenol down-regulation of IL-6/TNF-α), mitigating oxidative stress (e.g., carotenoid activation of the Nrf2 pathway (22)), and modulating gut microbiota (e.g., fiber-induced SCFA production enhancing insulin signaling), all of which are critical pathways in the pathophysiology of GDM (20, 21).

Compared with previous studies, our study offers several advantages. First, the large sample size allowed sufficient statistical power to detect associations across PDI quartiles and to adjust for multiple potential confounders. Second, dietary intake was assessed prospectively in early pregnancy before GDM diagnosis, minimizing reverse causation and recall bias. Third, we comprehensively adjusted for biochemical, sociodemographic, and lifestyle factors, including maternal supplement use, total energy intake, and physical activity, which strengthens the robustness of our findings. In addition, the inclusion of gestational outcomes, such as gestational hypertension, preeclampsia, preterm birth, and birthweight, revealed that higher PDI was associated with lower prevalence of adverse pregnancy events and slightly higher mean birthweight, highlighting broader perinatal benefits. Our PDI findings align with Mediterranean and DASH diets, which reduce GDM risk by 15–25% via similar plant-based components (13, 19); however, PDI’s focus on phytochemical proportion offers a novel, quantifiable metric for targeted interventions beyond broad patterns.

Mechanistically, the observed associations may be explained by the synergistic effects of multiple phytochemicals. Polyphenols and carotenoids reduce systemic inflammation by down-regulating proinflammatory cytokines (e.g., IL-6, TNF-α) and enhancing antioxidant pathways (22). Dietary fiber from fruit, vegetables, and whole grains improves postprandial glucose responses by slowing carbohydrate absorption and promoting satiety (23). Nuts and olive oil provide monounsaturated and polyunsaturated fatty acids that favorably influence lipid metabolism and insulin sensitivity (24). Collectively, these biological effects provide a plausible explanation not only for the inverse association between PDI and GDM, but also for the observed improvements in maternal lipid profiles and reduced risk of neonatal complications.

While our findings are generally consistent with the beneficial effects of phytochemical-rich diets, some prior studies reported weaker or non-significant associations, potentially due to smaller sample sizes, differences in dietary assessment methods, or variation in the timing of diet evaluation (5, 12, 14, 19). By assessing diet in early pregnancy, our study captures a critical window when maternal metabolic adaptations occur, supporting the importance of early dietary interventions. Moreover, our analysis showed that higher PDI was positively correlated with intake of fiber, whole grains, fruit, vegetables, legumes, nuts and olive oil, indicating that diet quality rather than total caloric intake drives these beneficial outcomes.

### Strengths and limitations

Strengths of this study include its prospective design, large and well-characterized cohort, standardized outcome assessment, and comprehensive adjustment for confounders. In addition, our analysis included both continuous and categorical PDI metrics, allowing evaluation of dose–response relationships across multiple biochemical and pregnancy outcomes. However, several limitations should be acknowledged. Dietary intake was self-reported and may be subject to recall bias, although FFQ validation reduces this concern. Residual confounding by unmeasured factors, such as genetic predisposition or environmental exposures, cannot be excluded. Although extensive adjustments were made for known confounders, residual confounding by unmeasured behavioral or genetic factors cannot be entirely excluded. Furthermore, self-reported dietary data may introduce minor reporting bias despite validation of the FFQ. Our findings are based on an urban Chinese population, which may limit generalizability to populations with different dietary habits. Finally, although we observed robust associations between higher PDI and improved maternal and neonatal outcomes, the observational design precludes causal inference, and randomized controlled trials are warranted to confirm these findings. In addition, as our cohort consisted of urban Chinese women with relatively homogeneous dietary patterns and moderate variation in habits, findings may not fully generalize to populations with diverse cultural, ethnic, or dietary backgrounds, and extrapolation should be made cautiously.

### Implications for practice and future research

Our findings highlight the potential of early-pregnancy dietary modification to reduce GDM risk and improve maternal and neonatal health. Encouraging intake of phytochemical-rich foods may represent a practical, safe, and cost-effective strategy to enhance metabolic and perinatal outcomes. Future research should investigate underlying biological mechanisms, including the roles of inflammation, oxidative stress, and gut microbiota, and evaluate targeted dietary interventions across diverse populations to confirm clinical benefits.

## Conclusions

In conclusion, higher consumption of phytochemical-rich foods in early pregnancy, reflected by elevated PDI scores, is associated with substantially lower risk of GDM, improved maternal biochemical profiles, reduced neonatal hypoglycemia, and more favorable gestational outcomes. These results reinforce the importance of integrating phytochemical-rich foods into prenatal nutrition guidance and provide a foundation for future interventional studies to prevent GDM and associated complications.

## Declaration of interest

The author declares that there is no conflict of interest that could be perceived as prejudicing the impartiality of the work reported.

## Funding

This work did not receive any specific grant from any funding agency in the public, commercial, or not-for-profit sector.

## Author contribution statement

Hngli Hou was responsible for conceptualization, methodology, supervision, and writing, review and editing.

## Data availability

All data generated or analyzed in this study are included in the manuscript. Additional data are available from the corresponding author upon reasonable request.

## Ethics approval and consent to participate

Approved by the Ethics Committee of the Children’s Hospital of Shanxi, Women’s Health Center of ShanXi (Approval Code: IRB-KY-2024-009). Written informed consent was obtained from all participants. All procedures complied with the Declaration of Helsinki.
